# Synthesis and structure of ethyl 2,2-dimethyl-3-(naphthalen-1-yl)-1,2-di­hydro­benzo[*c*]siline-4-carboxyl­ate

**DOI:** 10.1107/S2056989026008388

**Published:** 2026-09-03

**Authors:** Xiaoxiao Tang, Liyun Zhang

**Affiliations:** a Third Hospital of Shanxi Medical University Shanxi Bethune Hospital Shanxi Academy of Medical Sciences, Tongji Shanxi Hospital Taiyuan 030032 People’s Republic of China; University of Aberdeen, United Kingdom

**Keywords:** crystal structure, silacycles, hydrogen bonds, extended π-conjugation

## Abstract

In the title compound, C_24_H_24_O_2_Si, the six-membered silacyclic ring adopts a twisted boat conformation and the naphthyl substituent is nearly orthogonal to the plane of the benzo[*c*]siline fused framework. The terminal ethyl ester fragment displays two-site static disorder. In the extended structure, weak C—H⋯O hydrogen bonds link adjacent mol­ecules into infinite zigzag ribbons propagating parallel to the [010] direction.

## Abstract 2

In the title compound, C_24_H_24_O_2_Si, the ethyl ester substituent shows two-site disorder and C—H⋯O contacts generate zigzag ribbons extending along [010].

## 1. Chemical context

Silacyclic fused skeletons represent appealing building blocks for synthetic chemistry, optoelectronic materials and medicinal chemistry (Bains *et al.*, 2003 ▸; Ikeno *et al.*, 2017 ▸; Fujii *et al.*, 2017 ▸). Silicon possesses a larger covalent radius, elongated Si—C bonds and accessible σ*–π* conjugation, which enables precise modulation of electronic and biological properties (Franz *et al.*, 2013 ▸; Gately *et al.*, 2007 ▸; Meanwell *et al.*, 2011 ▸). Silicon isosteres can simultaneously enhance mol­ecular lipophilicity and chemical stability (Min *et al.*, 2013 ▸; Rémond *et al.*, 2016 ▸). Conjugated six-membered benzo[*c*]silines feature rigid and readily tunable almost planar backbones, yet synthetic and crystallographic investigations of functionalized derivatives lag significantly behind those of five-membered silole analogues (Santra, 2020 ▸).

The parent 1,2-di­hydro­benzo[*c*]siline framework features an *sp*^3^-hybridized tetra­hedral silicon atom. Endocyclic alkene, aryl and ester substituents can establish multiple intra­molecular conjugation pathways. Functionalization with *gem*-dimethyl, 1-naphthyl and ethyl carboxyl­ate groups extends the effective π-conjugation length; steric hindrance originating from the *gem*-dimethyl substituents modulates ring geometry, and the naphthalene moiety provides abundant sites for inter­molecular π-related inter­actions. To date, no single-crystal X-ray diffraction data have been documented for this triple-substituted scaffold, leaving its solid-state mol­ecular conformation and supra­molecular packing unexplored. Ring expansion of benzosila­cyclo­butene provides a facile, atom-economic synthetic route to functionalized benzo[*c*]silines (Wang *et al.*, 2020 ▸; Wang *et al.*, 2025 ▸).

**Figure 1:**
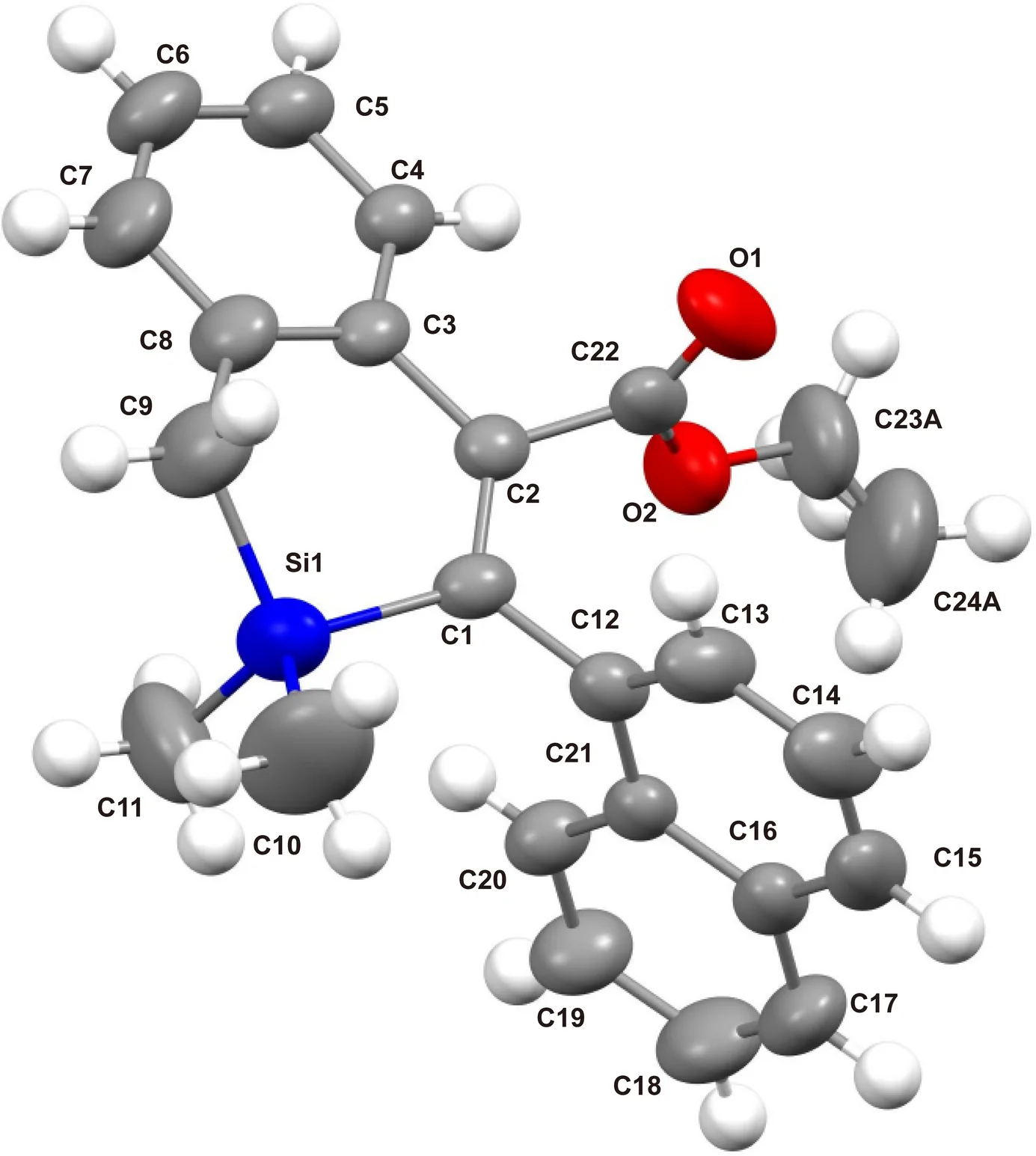
The mol­ecular structure of **3** with displacement ellipsoids drawn at the 50% probability level. Only the major disorder component of the ester side chain is shown; hydrogen atoms are omitted for clarity.

As part of our studies in this area, we now describe the synthesis and structure of the title compound, C_24_H_24_O_2_Si (**3**) (Fig. 1 ▸).
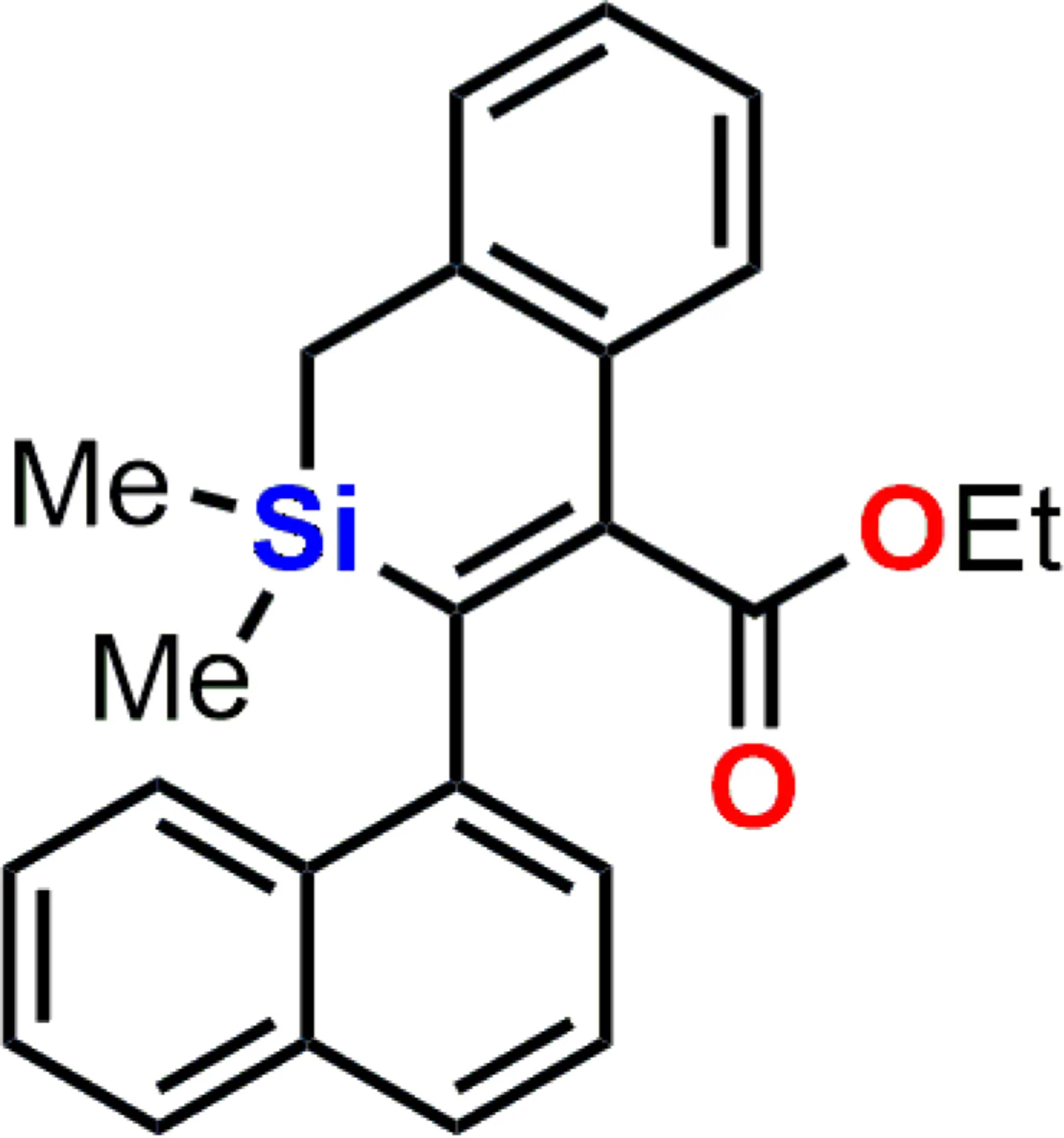


## 2. Structural commentary

The tetra­hedral silicon atom Si1 has four distinct Si—C single bonds ranging from 1.847 (3) to 1.879 (2) Å; the longest Si1—C1 bond corresponds to the olefin-linked carbon atom, which leads to a narrow C9—Si1—C1 angle of 100.76 (11)°, producing obvious puckering of the silacyclic ring. The C1=C2 double bond at 1.340 (3) Å is the shortest carbon–carbon linkage within the whole skeleton, consistent with the characteristic length of C=C olefin bonds. The six-membered silacyclic ring adopts a twisted boat conformation with C2/C3/C8/C9 almost coplanar (r.m.s. deviation = 0.010 Å) and Si1 and C1 displaced from their mean plane by 0.947 (1) and 0.437 (2) Å, respectively. The torsion angle Si1—C1—C2—C3 of −5.4 (3)° confirms minor deviation of the unsaturated segment from the ring mean plane. The fused benzene moiety on the silacycle remains nearly planar, with the C3—C8—C9 bond angle of 121.5 (2)° matching standard aromatic C—C—C angles. The torsion angle C3—C8—C9—Si1 = −36.2 (3)° qu­anti­fies the out-of-plane displacement of the Si atom relative to the benzo-fused plane.

A single C1—C12 σ-bond links the silacyclic core and pendant naphthalene ring. The large torsion angle Si1—C1—C12—C13 = −93.6 (2)° reveals that the naphthalene plane is nearly perpendicular to the benzosiline plane, a result of steric repulsion from the *gem*-dimethyl substituents on silicon, which restricts intra­molecular π-conjugation between the two aromatic systems. The naphthalene fragment maintains ideal aromatic geometry, exemplified by the C16—C21—C12 bond angle of 119.7 (2)°.

At the C2 position of the silacycle lies an ethyl carboxyl­ate group. The carbonyl bond C22=O1 [1.195 (3) Å] and ester single bond C22—O2 [1.320 (3) Å] show typical carbon­yl/ether bond lengths for carboxyl­ate esters. The bond angle O1—C22—O2 reaches 124.8 (3)°, while O2—C22—C2 is 111.2 (2)°, reflecting the *sp*^2^ hybridization state of carbonyl carbon atom C22. The terminal ethyl group on O2 suffers two-site static disorder, as demonstrated by the torsion angle C22—O2—C23—C24 of 101.7 (10)° for one disordered alkyl conformation; the other disordered ethyl chain adopts a separate rotational orientation with different torsion value. Apart from the disordered eth­oxy alkyl chain, all rigid aromatic and silacyclic fragments display normal bond lengths and angles without significant structural distortion.

## 3. Supra­molecular features

**Figure 2:**
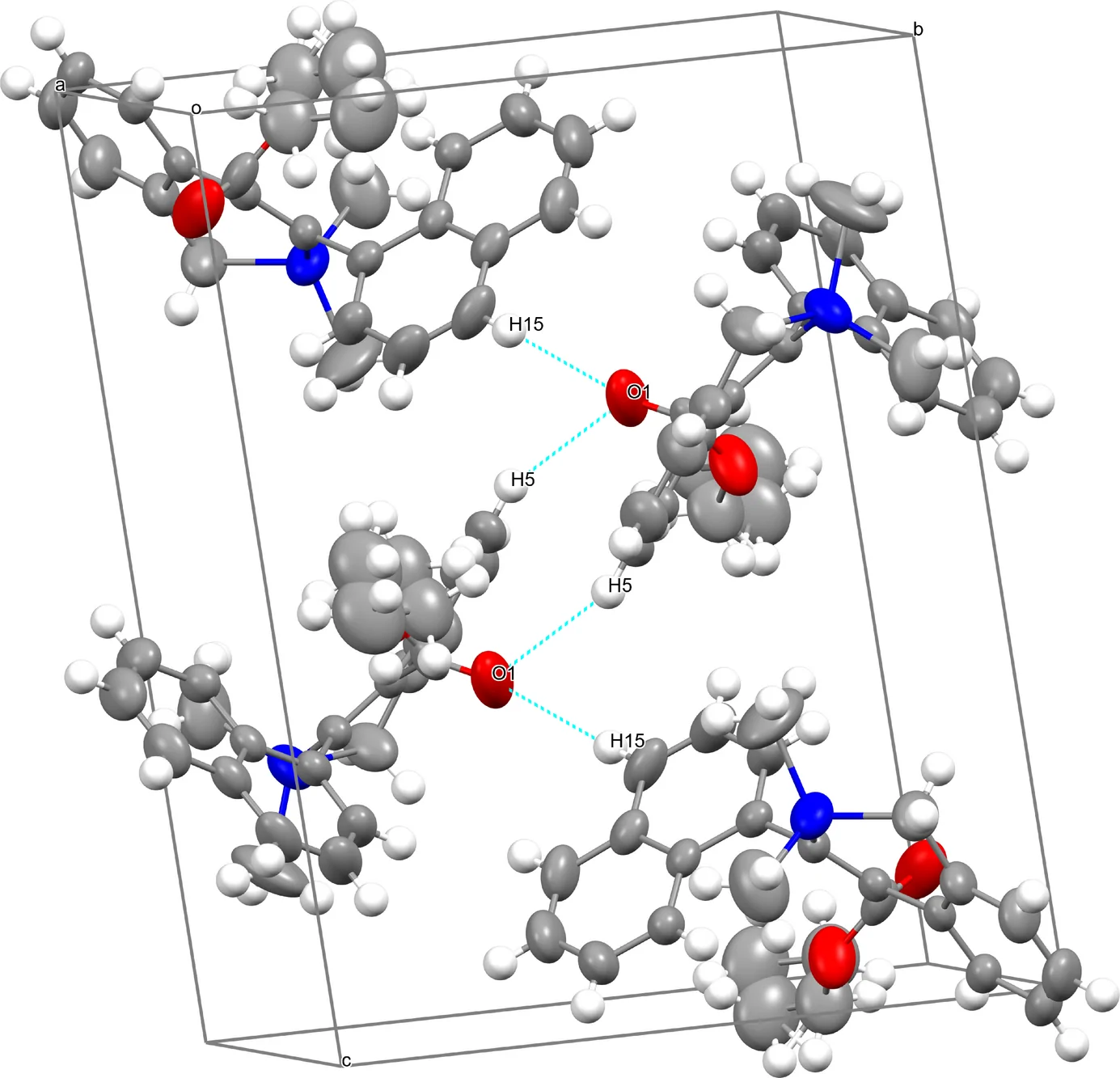
Crystal packing diagram for **3** with hydrogen bonds represented by dashed lines.

In the extended structure of **3**, weak C15—H15⋯O1 hydrogen bonds link the mol­ecules into infinite zigzag ribbons propagating parallel to the [010] crystallographic direction (Fig. 2 ▸). Geometric parameters for this inter­action are listed in Table 1 ▸.

## 4. Database survey

Searches were carried out with the Cambridge Structural Database(CSD, Version 6.01, update November 2025; Groom *et al.*, 2016 ▸) together with the *ConQuest* search program (Version 2025.3.1; Bruno *et al.*, 2002 ▸).

An unrestricted search for the parent 1,2-di­hydro­benzo[*c*]siline fused silacyclic core without extra fused aromatic rings returned a moderate number of hits, most of which carry simple alkyl or phenyl substituents only (Wang *et al.*, 2026 ▸). Limiting the query to 2,2-dimethyl-substituted 1,2-di­hydro­benzo[*c*]siline skeletons yielded far fewer recorded structures, indicating this di­methyl­silacyclic scaffold remains underexplored in single-crystal crystallography.

Further constraints were applied to narrow the screening: the silacycle bears an ethyl carboxyl­ate group at the C2 position and a pendant naphthalen-1-yl group attached to the ring olefinic carbon atom. After applying this triple substitution filter (*gem*-dimethyl silicon, 4-carboxyl­ate ester, 3-naphth­yl), zero matching crystal structures were retrieved from the CSD.

The few reported analogues of 1,2-di­hydro­benzo[*c*]siline only incorporate small monocyclic aryl groups such as phenyl, rather than extended polycyclic naphthalene fragments. A supplementary restricted search was performed for carboxyl­ate-functionalized benzosilacyclic mol­ecules without naphthyl substitution. Only a few simple phenyl-substituted ethyl benzo[*c*]siline carboxyl­ate entries were found (Tang *et al.*, 2022 ▸; Tang *et al.*, 2024 ▸), none of which feature the bulky naphthalene π-extended moiety on the silacyclic backbone.

## 5. Synthesis and crystallization

**Figure 3:**
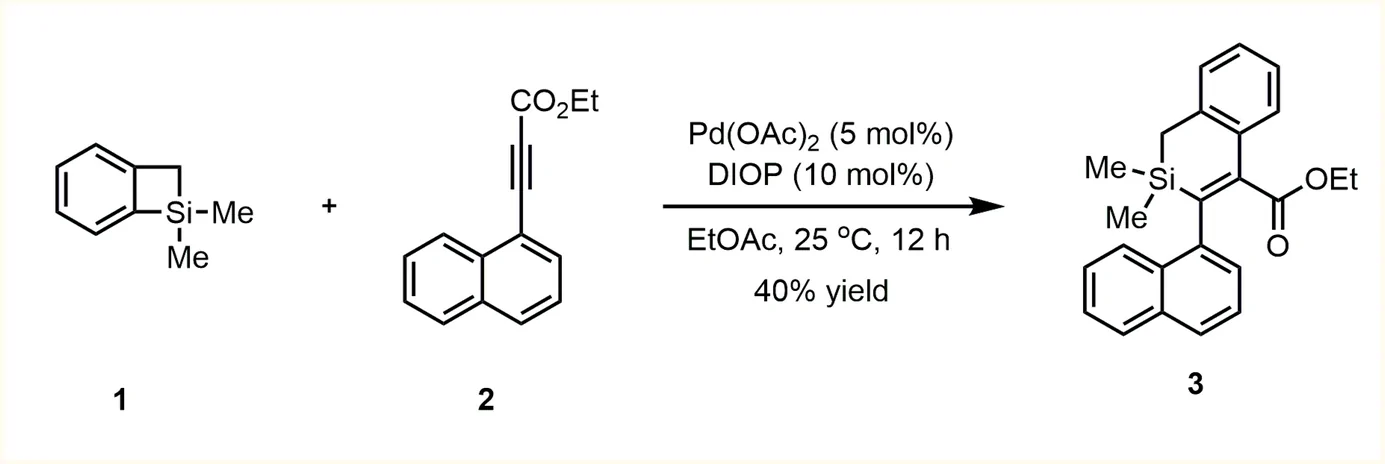
The synthesis of compound **3**.

The synthetic route for the title compound is shown in Fig. 3 ▸. A solution of Pd(OAc)_2_ (2.2 mg, 0.01 mmol, 5 mol %) and [(2,2-dimethyl-1,3-dioxolane-4,5-diyl)dimethanediyl]bis(diphen­ylphosphane (DIOP) (10.0 mg, 0.02 mmol, 10 mol %) in EtOAc (0.5 ml) was stirred at 298 K for 3 min. Alkyne **2** (44.8 mg, 0.2 mmol, 1.0 equiv.), benzosila­cyclo­butene **1** (59.3 mg, 0.4 mmol, 2.0 equiv.) and additional EtOAc (1.5 ml) were added sequentially. The mixture was kept at 298 K for 12 h. The solvent was removed under reduced pressure, and the residue was purified by silica gel column chromatography with gradient elution (petroleum ether, then petroleum ether/ethyl acetate = 50:1) to furnish the target product **3** (yield: 30 mg, 40%). ^1^H NMR (400 MHz, CDCl_3_) δ 7.88–7.82 (*m*, 2H), 7.71 (*d*, *J* = 8.3 Hz, 1H), 7.47–7.39 (*m*, 4H), 7.21–7.12 (*m*, 4H), 3.71 (*q*, *J* = 7.1, 2H), 2.36 (*s*, 2H), 0.45 (*t*, *J* = 7.1 Hz, 3H), 0.03 (*s*, 3H), −0.01 (*s*, 3H). A solution of **3** (20 mg) in 2.0 ml mixed solvent of CH_2_Cl_2_/*n*-hexane (CH_2_Cl_2_:*n*-hexane = 1:3) was transferred to a test tube and sealed with plastic film. Suitable single crystals of **3** were obtained by slow solvent evaporation over 3 days at room temperature.

## 6. Refinement

Details of data collection and structure refinement are summarized in Table 2 ▸. The terminal ethyl group of the ester moiety exhibits two-site static positional disorder. Two sets of atomic coordinates (C23/C24 and C23*A*/C24*A*) were modelled for the disordered alkyl fragment sharing the ether oxygen atom O2. Suitable distance restraints and anisotropic displacement parameter constraints were adopted to achieve stable refinement of the disordered part. All H atoms were included using a riding model starting from calculated positions. Methyl groups were refined as idealized rigid groups, with C—H = 0.96 Å and H—C—H = 109.5°, allowed to rotate but not tip (AFIX 137 card in *SHELXL*). Methyl­ene and aromatic H atoms were constrained with C—H = 0.97 Å and 0.93 Å, respectively. The *U*_iso_(H) values were fixed at 1.5U_eq_ of the parent C atoms for methyl groups and at 1.2U_eq_ for the remaining H atoms.

## Supplementary Material

Crystal structure: contains datablock(s) I. DOI: 10.1107/S2056989026008388/hb8244sup1.cif

Structure factors: contains datablock(s) I. DOI: 10.1107/S2056989026008388/hb8244Isup2.hkl

Supporting information file. DOI: 10.1107/S2056989026008388/hb8244Isup3.cml

CCDC reference: 2575735

Additional supporting information:  crystallographic information; 3D view; checkCIF report

## Figures and Tables

**Table 1:** Hydrogen-bond geometry (Å, °)

*D*—H⋯*A*	*D*—H	H⋯*A*	*D*⋯*A*	*D*—H⋯*A*
C15—H15⋯O1^i^	0.93	2.49	3.359 (4)	156

Symmetry code: (i) 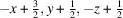.

**Table 2:** Experimental details

Crystal data	
Chemical formula	C_24_H_24_O_2_Si
*M*_r_	372.52
Crystal system, space group	Monoclinic, *P*2_1_/*n*
Temperature (K)	293
*a*, *b*, *c* (Å)	9.8031 (5), 13.0620 (6), 17.1604 (10)
β (°)	105.412 (6)
*V* (Å^3^)	2118.3 (2)
*Z*	4
*D_x_* (Mg m^−3^)	1.168
Radiation type	Mo *K*α
μ (mm^−1^)	0.13
Crystal size (mm)	0.35 × 0.30 × 0.25

Data collection	
Diffractometer	Xcalibur, Eos
Absorption correction	Multi-scan (*CrysAlis PRO*; Agilent, 2014 ▸)
*T*_min_, *T*_max_	0.977, 1.000
No. of measured, independent and observed [*I* > 2σ(*I*)] reflections	9418, 4331, 2739
*R*_int_	0.027
(sin θ/λ)_max_ (Å^−1^)	0.625

Refinement	
*R*[*F*^2^ > 2σ(*F*^2^)], *wR*(*F*^2^), *S*	0.057, 0.144, 1.02
No. of reflections	4331
No. of parameters	255
No. of restraints	2
H-atom treatment	H-atom parameters constrained
Δρ_max_, Δρ_min_ (e Å^−3^)	0.20, −0.22

Computer programs: *CrysAlis PRO* (Agilent, 2014 ▸), *OLEX2.solve* (Bourhis *et al.*, 2015 ▸), *SHELXL* (Sheldrick, 2015 ▸) and *OLEX2* (Dolomanov *et al.*, 2009 ▸).

## supplementary crystallographic information

### Ethyl 2,2-dimethyl-3-(naphthalen-1-yl)-1,2-dihydrobenzo[c]siline-4-carboxylate Crystal data

**Table:**

C_24_H_24_O_2_Si	*F*(000) = 792
*M**_r_* = 372.52	*D*_x_ = 1.168 Mg m^−^^3^
Monoclinic, *P*2_1_/*n*	Mo *K*α radiation, λ = 0.71073 Å
*a* = 9.8031 (5) Å	Cell parameters from 2164 reflections
*b* = 13.0620 (6) Å	θ = 3.8–24.9°
*c* = 17.1604 (10) Å	µ = 0.13 mm^−^^1^
β = 105.412 (6)°	*T* = 293 K
*V* = 2118.3 (2) Å^3^	Block, colourless
*Z* = 4	0.35 × 0.30 × 0.25 mm

### Ethyl 2,2-dimethyl-3-(naphthalen-1-yl)-1,2-dihydrobenzo[c]siline-4-carboxylate Data collection

**Table:**

Xcalibur, Eos diffractometer	4331 independent reflections
Radiation source: Enhance (Mo) X-ray Source	2739 reflections with *I* > 2σ(*I*)
Graphite monochromator	*R*_int_ = 0.027
Detector resolution: 16.0874 pixels mm^-1^	θ_max_ = 26.4°, θ_min_ = 3.1°
ω scans	*h* = −12→11
Absorption correction: multi-scan (CrysalisPro; Agilent, 2014)	*k* = −10→16
*T*_min_ = 0.977, *T*_max_ = 1.000	*l* = −21→21
9418 measured reflections	

### Ethyl 2,2-dimethyl-3-(naphthalen-1-yl)-1,2-dihydrobenzo[c]siline-4-carboxylate Refinement

**Table:**

Refinement on *F*^2^	Primary atom site location: dual
Least-squares matrix: full	Hydrogen site location: inferred from neighbouring sites
*R*[*F*^2^ > 2σ(*F*^2^)] = 0.057	H-atom parameters constrained
*wR*(*F*^2^) = 0.144	*w* = 1/[σ^2^(*F*_o_^2^) + (0.0533*P*)^2^ + 0.4704*P*] where *P* = (*F*_o_^2^ + 2*F*_c_^2^)/3
*S* = 1.02	(Δ/σ)_max_ = 0.005
4331 reflections	Δρ_max_ = 0.20 e Å^−^^3^
255 parameters	Δρ_min_ = −0.22 e Å^−^^3^
2 restraints	

### Ethyl 2,2-dimethyl-3-(naphthalen-1-yl)-1,2-dihydrobenzo[c]siline-4-carboxylate Special details

**Table:**

Geometry. All esds (except the esd in the dihedral angle between two l.s. planes) are estimated using the full covariance matrix. The cell esds are taken into account individually in the estimation of esds in distances, angles and torsion angles; correlations between esds in cell parameters are only used when they are defined by crystal symmetry. An approximate (isotropic) treatment of cell esds is used for estimating esds involving l.s. planes.

### Ethyl 2,2-dimethyl-3-(naphthalen-1-yl)-1,2-dihydrobenzo[c]siline-4-carboxylate Fractional atomic coordinates and isotropic or equivalent isotropic displacement parameters (Å^2^)

**Table:**

	*x*	*y*	*z*	*U*_iso_*/*U*_eq_	Occ. (<1)
Si1	0.19934 (7)	0.86055 (6)	0.28273 (5)	0.0677 (3)	
O1	0.6312 (2)	0.64582 (15)	0.36738 (13)	0.0948 (7)	
O2	0.65900 (17)	0.78080 (15)	0.44955 (13)	0.0849 (6)	
C1	0.3927 (2)	0.82910 (15)	0.32118 (12)	0.0451 (5)	
C2	0.4304 (2)	0.74794 (15)	0.36995 (12)	0.0414 (5)	
C3	0.3349 (2)	0.68302 (15)	0.40340 (12)	0.0432 (5)	
C4	0.3930 (2)	0.62571 (15)	0.47252 (13)	0.0496 (6)	
H4	0.490195	0.628386	0.495618	0.060*	
C5	0.3114 (3)	0.56525 (18)	0.50777 (16)	0.0618 (7)	
H5	0.352714	0.528183	0.554331	0.074*	
C6	0.1692 (3)	0.5602 (2)	0.47376 (19)	0.0760 (8)	
H6	0.113043	0.519214	0.496946	0.091*	
C7	0.1087 (3)	0.6156 (2)	0.40530 (19)	0.0766 (8)	
H7	0.011395	0.611590	0.382979	0.092*	
C8	0.1887 (2)	0.67750 (18)	0.36838 (15)	0.0585 (6)	
C9	0.1192 (3)	0.7340 (2)	0.29122 (18)	0.0804 (8)	
H9A	0.019767	0.743510	0.288184	0.096*	
H9B	0.125079	0.692078	0.245566	0.096*	
C10	0.1588 (4)	0.9036 (3)	0.1761 (2)	0.1390 (16)	
H10A	0.209245	0.965751	0.172977	0.208*	
H10B	0.058954	0.915557	0.156109	0.208*	
H10C	0.187152	0.851655	0.144024	0.208*	
C11	0.1435 (3)	0.9572 (3)	0.3466 (2)	0.1177 (13)	
H11A	0.174468	0.936650	0.402202	0.177*	
H11B	0.042264	0.963050	0.330741	0.177*	
H11C	0.184829	1.022193	0.339953	0.177*	
C12	0.5024 (2)	0.88483 (16)	0.29121 (13)	0.0480 (5)	
C13	0.5465 (3)	0.84581 (19)	0.22820 (15)	0.0684 (7)	
H13	0.506983	0.785008	0.204275	0.082*	
C14	0.6496 (3)	0.8948 (2)	0.19860 (18)	0.0820 (9)	
H14	0.677997	0.866046	0.155836	0.098*	
C15	0.7083 (3)	0.9840 (2)	0.23209 (18)	0.0741 (8)	
H15	0.776477	1.016121	0.211939	0.089*	
C16	0.6667 (2)	1.02835 (18)	0.29737 (15)	0.0556 (6)	
C17	0.7270 (3)	1.1201 (2)	0.3343 (2)	0.0747 (8)	
H17	0.796146	1.152936	0.315451	0.090*	
C18	0.6855 (3)	1.1609 (2)	0.3966 (2)	0.0817 (8)	
H18	0.726806	1.221327	0.420416	0.098*	
C19	0.5817 (3)	1.11345 (19)	0.42555 (16)	0.0702 (7)	
H19	0.553016	1.142822	0.467961	0.084*	
C20	0.5221 (2)	1.02434 (17)	0.39214 (13)	0.0536 (6)	
H20	0.454311	0.992613	0.412859	0.064*	
C21	0.5612 (2)	0.97902 (16)	0.32639 (13)	0.0454 (5)	
C22	0.5840 (3)	0.7176 (2)	0.39466 (16)	0.0576 (6)	
C23	0.8087 (12)	0.7740 (13)	0.4996 (10)	0.101 (3)	0.445 (11)
H23A	0.844249	0.704576	0.500442	0.121*	0.445 (11)
H23B	0.817204	0.796147	0.554594	0.121*	0.445 (11)
C24	0.8871 (15)	0.8457 (13)	0.4572 (10)	0.143 (4)	0.555 (11)
H24A	0.862779	0.830092	0.400473	0.214*	0.555 (11)
H24B	0.987223	0.837422	0.479513	0.214*	0.555 (11)
H24C	0.861109	0.915184	0.464637	0.214*	0.555 (11)
C23A	0.8130 (9)	0.7548 (10)	0.4550 (9)	0.101 (3)	0.555 (11)
H23C	0.839613	0.688670	0.480229	0.121*	0.555 (11)
H23D	0.831899	0.755889	0.402343	0.121*	0.555 (11)
C24A	0.8855 (19)	0.8400 (16)	0.5071 (12)	0.143 (4)	0.445 (11)
H24D	0.855128	0.904124	0.480903	0.214*	0.445 (11)
H24E	0.986018	0.833151	0.515975	0.214*	0.445 (11)
H24F	0.862360	0.837720	0.557994	0.214*	0.445 (11)

### Ethyl 2,2-dimethyl-3-(naphthalen-1-yl)-1,2-dihydrobenzo[c]siline-4-carboxylate Atomic displacement parameters (Å^2^)

**Table:**

	*U*^11^	*U*^22^	*U*^33^	*U*^12^	*U*^13^	*U*^23^
Si1	0.0569 (4)	0.0664 (5)	0.0688 (5)	−0.0029 (4)	−0.0025 (3)	0.0175 (4)
O1	0.1178 (17)	0.0744 (13)	0.1161 (17)	0.0434 (12)	0.0727 (14)	0.0215 (12)
O2	0.0432 (9)	0.0742 (13)	0.1242 (17)	0.0014 (9)	−0.0006 (10)	0.0113 (13)
C1	0.0577 (12)	0.0368 (11)	0.0410 (12)	−0.0052 (10)	0.0137 (10)	0.0001 (10)
C2	0.0500 (12)	0.0357 (11)	0.0403 (11)	−0.0018 (10)	0.0150 (9)	−0.0035 (10)
C3	0.0527 (12)	0.0345 (10)	0.0450 (12)	−0.0055 (10)	0.0172 (10)	−0.0056 (10)
C4	0.0584 (13)	0.0425 (12)	0.0517 (14)	−0.0019 (11)	0.0211 (11)	0.0013 (11)
C5	0.0782 (17)	0.0507 (14)	0.0620 (16)	−0.0047 (13)	0.0282 (14)	0.0082 (12)
C6	0.0823 (19)	0.0629 (17)	0.092 (2)	−0.0192 (15)	0.0395 (17)	0.0083 (16)
C7	0.0566 (15)	0.0706 (17)	0.100 (2)	−0.0222 (14)	0.0162 (15)	0.0036 (17)
C8	0.0569 (13)	0.0512 (13)	0.0629 (16)	−0.0152 (12)	0.0080 (12)	−0.0026 (12)
C9	0.0619 (15)	0.085 (2)	0.0791 (19)	−0.0241 (15)	−0.0075 (13)	0.0090 (17)
C10	0.115 (3)	0.174 (4)	0.101 (3)	−0.007 (3)	−0.017 (2)	0.077 (3)
C11	0.0648 (19)	0.107 (3)	0.177 (4)	0.0185 (19)	0.023 (2)	−0.015 (3)
C12	0.0583 (13)	0.0437 (12)	0.0452 (13)	0.0066 (11)	0.0193 (10)	0.0080 (11)
C13	0.102 (2)	0.0504 (14)	0.0643 (16)	0.0039 (14)	0.0426 (16)	0.0048 (13)
C14	0.117 (2)	0.0681 (18)	0.084 (2)	0.0192 (18)	0.0670 (19)	0.0151 (16)
C15	0.0771 (18)	0.0685 (18)	0.094 (2)	0.0139 (15)	0.0525 (17)	0.0324 (17)
C16	0.0522 (13)	0.0528 (14)	0.0653 (16)	0.0071 (12)	0.0216 (12)	0.0199 (13)
C17	0.0608 (16)	0.0620 (17)	0.102 (2)	−0.0156 (14)	0.0221 (16)	0.0156 (17)
C18	0.085 (2)	0.0609 (17)	0.093 (2)	−0.0248 (16)	0.0127 (17)	−0.0042 (17)
C19	0.0853 (18)	0.0589 (16)	0.0651 (17)	−0.0166 (15)	0.0175 (14)	−0.0089 (14)
C20	0.0617 (13)	0.0500 (13)	0.0501 (13)	−0.0084 (12)	0.0168 (11)	0.0024 (12)
C21	0.0453 (11)	0.0420 (12)	0.0490 (13)	0.0035 (10)	0.0126 (10)	0.0117 (10)
C22	0.0649 (15)	0.0490 (14)	0.0698 (17)	0.0099 (13)	0.0369 (14)	0.0225 (13)
C23	0.0436 (18)	0.111 (5)	0.143 (9)	0.006 (3)	0.016 (5)	0.006 (7)
C24	0.063 (2)	0.168 (5)	0.196 (14)	−0.014 (3)	0.032 (9)	0.021 (11)
C23A	0.0436 (18)	0.111 (5)	0.143 (9)	0.006 (3)	0.016 (5)	0.006 (7)
C24A	0.063 (2)	0.168 (5)	0.196 (14)	−0.014 (3)	0.032 (9)	0.021 (11)

### Ethyl 2,2-dimethyl-3-(naphthalen-1-yl)-1,2-dihydrobenzo[c]siline-4-carboxylate Geometric parameters (Å, º)

**Table:**

Si1—C1	1.879 (2)	C12—C13	1.365 (3)
Si1—C9	1.853 (3)	C12—C21	1.423 (3)
Si1—C10	1.853 (3)	C13—H13	0.9300
Si1—C11	1.847 (3)	C13—C14	1.401 (3)
O1—C22	1.195 (3)	C14—H14	0.9300
O2—C22	1.320 (3)	C14—C15	1.358 (4)
O2—C23	1.493 (12)	C15—H15	0.9300
O2—C23A	1.526 (9)	C15—C16	1.414 (3)
C1—C2	1.340 (3)	C16—C17	1.409 (4)
C1—C12	1.498 (3)	C16—C21	1.416 (3)
C2—C3	1.487 (3)	C17—H17	0.9300
C2—C22	1.505 (3)	C17—C18	1.351 (4)
C3—C4	1.390 (3)	C18—H18	0.9300
C3—C8	1.401 (3)	C18—C19	1.391 (4)
C4—H4	0.9300	C19—H19	0.9300
C4—C5	1.374 (3)	C19—C20	1.359 (3)
C5—H5	0.9300	C20—H20	0.9300
C5—C6	1.362 (4)	C20—C21	1.415 (3)
C6—H6	0.9300	C23—H23A	0.9700
C6—C7	1.373 (4)	C23—H23B	0.9700
C7—H7	0.9300	C23—C24	1.516 (17)
C7—C8	1.391 (3)	C24—H24A	0.9600
C8—C9	1.511 (3)	C24—H24B	0.9600
C9—H9A	0.9700	C24—H24C	0.9600
C9—H9B	0.9700	C23A—H23C	0.9700
C10—H10A	0.9600	C23A—H23D	0.9700
C10—H10B	0.9600	C23A—C24A	1.484 (16)
C10—H10C	0.9600	C24A—H24D	0.9600
C11—H11A	0.9600	C24A—H24E	0.9600
C11—H11B	0.9600	C24A—H24F	0.9600
C11—H11C	0.9600		
			
C9—Si1—C1	100.76 (11)	C12—C13—C14	121.9 (3)
C9—Si1—C10	111.35 (17)	C14—C13—H13	119.1
C10—Si1—C1	110.16 (15)	C13—C14—H14	119.9
C11—Si1—C1	111.79 (12)	C15—C14—C13	120.3 (2)
C11—Si1—C9	111.38 (16)	C15—C14—H14	119.9
C11—Si1—C10	111.0 (2)	C14—C15—H15	119.7
C22—O2—C23	130.5 (7)	C14—C15—C16	120.5 (2)
C22—O2—C23A	105.0 (5)	C16—C15—H15	119.7
C2—C1—Si1	118.51 (15)	C15—C16—C21	118.9 (2)
C2—C1—C12	119.63 (19)	C17—C16—C15	122.0 (2)
C12—C1—Si1	121.48 (15)	C17—C16—C21	119.1 (2)
C1—C2—C3	126.47 (19)	C16—C17—H17	119.6
C1—C2—C22	118.28 (18)	C18—C17—C16	120.9 (2)
C3—C2—C22	115.25 (18)	C18—C17—H17	119.6
C4—C3—C2	118.63 (19)	C17—C18—H18	119.7
C4—C3—C8	118.6 (2)	C17—C18—C19	120.6 (3)
C8—C3—C2	122.8 (2)	C19—C18—H18	119.7
C3—C4—H4	119.0	C18—C19—H19	119.8
C5—C4—C3	122.0 (2)	C20—C19—C18	120.3 (3)
C5—C4—H4	119.0	C20—C19—H19	119.8
C4—C5—H5	120.4	C19—C20—H20	119.4
C6—C5—C4	119.2 (2)	C19—C20—C21	121.1 (2)
C6—C5—H5	120.4	C21—C20—H20	119.4
C5—C6—H6	119.9	C16—C21—C12	119.7 (2)
C5—C6—C7	120.1 (2)	C20—C21—C12	122.32 (19)
C7—C6—H6	119.9	C20—C21—C16	117.9 (2)
C6—C7—H7	119.1	O1—C22—O2	124.8 (3)
C6—C7—C8	121.9 (2)	O1—C22—C2	124.0 (3)
C8—C7—H7	119.1	O2—C22—C2	111.2 (2)
C3—C8—C9	121.5 (2)	O2—C23—H23A	111.1
C7—C8—C3	118.1 (2)	O2—C23—H23B	111.1
C7—C8—C9	120.4 (2)	O2—C23—C24	103.5 (10)
Si1—C9—H9A	108.8	H23A—C23—H23B	109.0
Si1—C9—H9B	108.8	C24—C23—H23A	111.1
C8—C9—Si1	113.76 (17)	C24—C23—H23B	111.1
C8—C9—H9A	108.8	C23—C24—H24A	109.5
C8—C9—H9B	108.8	C23—C24—H24B	109.5
H9A—C9—H9B	107.7	C23—C24—H24C	109.5
Si1—C10—H10A	109.5	H24A—C24—H24B	109.5
Si1—C10—H10B	109.5	H24A—C24—H24C	109.5
Si1—C10—H10C	109.5	H24B—C24—H24C	109.5
H10A—C10—H10B	109.5	O2—C23A—H23C	111.7
H10A—C10—H10C	109.5	O2—C23A—H23D	111.7
H10B—C10—H10C	109.5	H23C—C23A—H23D	109.5
Si1—C11—H11A	109.5	C24A—C23A—O2	100.3 (10)
Si1—C11—H11B	109.5	C24A—C23A—H23C	111.7
Si1—C11—H11C	109.5	C24A—C23A—H23D	111.7
H11A—C11—H11B	109.5	C23A—C24A—H24D	109.5
H11A—C11—H11C	109.5	C23A—C24A—H24E	109.5
H11B—C11—H11C	109.5	C23A—C24A—H24F	109.5
C13—C12—C1	119.8 (2)	H24D—C24A—H24E	109.5
C13—C12—C21	118.7 (2)	H24D—C24A—H24F	109.5
C21—C12—C1	121.43 (18)	H24E—C24A—H24F	109.5
C12—C13—H13	119.1		
			
Si1—C1—C2—C3	−5.4 (3)	C10—Si1—C9—C8	158.9 (2)
Si1—C1—C2—C22	174.72 (17)	C11—Si1—C1—C2	94.9 (2)
Si1—C1—C12—C13	−93.6 (2)	C11—Si1—C1—C12	−92.2 (2)
Si1—C1—C12—C21	85.7 (2)	C11—Si1—C9—C8	−76.6 (2)
C1—Si1—C9—C8	42.1 (2)	C12—C1—C2—C3	−178.53 (19)
C1—C2—C3—C4	−158.4 (2)	C12—C1—C2—C22	1.6 (3)
C1—C2—C3—C8	21.3 (3)	C12—C13—C14—C15	−0.4 (4)
C1—C2—C22—O1	−104.5 (3)	C13—C12—C21—C16	−2.0 (3)
C1—C2—C22—O2	75.1 (3)	C13—C12—C21—C20	−179.6 (2)
C1—C12—C13—C14	−179.5 (2)	C13—C14—C15—C16	0.3 (4)
C1—C12—C21—C16	178.78 (19)	C14—C15—C16—C17	179.1 (3)
C1—C12—C21—C20	1.2 (3)	C14—C15—C16—C21	−1.0 (4)
C2—C1—C12—C13	79.3 (3)	C15—C16—C17—C18	−179.9 (3)
C2—C1—C12—C21	−101.5 (2)	C15—C16—C21—C12	1.8 (3)
C2—C3—C4—C5	178.90 (19)	C15—C16—C21—C20	179.5 (2)
C2—C3—C8—C7	−179.0 (2)	C16—C17—C18—C19	−0.3 (4)
C2—C3—C8—C9	3.0 (4)	C17—C16—C21—C12	−178.2 (2)
C3—C2—C22—O1	75.7 (3)	C17—C16—C21—C20	−0.5 (3)
C3—C2—C22—O2	−104.8 (2)	C17—C18—C19—C20	1.0 (4)
C3—C4—C5—C6	0.7 (4)	C18—C19—C20—C21	−1.4 (4)
C3—C8—C9—Si1	−36.2 (3)	C19—C20—C21—C12	178.8 (2)
C4—C3—C8—C7	0.7 (3)	C19—C20—C21—C16	1.2 (3)
C4—C3—C8—C9	−177.3 (2)	C21—C12—C13—C14	1.3 (4)
C4—C5—C6—C7	−0.4 (4)	C21—C16—C17—C18	0.1 (4)
C5—C6—C7—C8	0.3 (4)	C22—O2—C23—C24	101.7 (10)
C6—C7—C8—C3	−0.5 (4)	C22—O2—C23A—C24A	171.2 (8)
C6—C7—C8—C9	177.5 (3)	C22—C2—C3—C4	21.4 (3)
C7—C8—C9—Si1	145.8 (2)	C22—C2—C3—C8	−158.9 (2)
C8—C3—C4—C5	−0.8 (3)	C23—O2—C22—O1	−9.8 (9)
C9—Si1—C1—C2	−23.5 (2)	C23—O2—C22—C2	170.7 (8)
C9—Si1—C1—C12	149.41 (18)	C23A—O2—C22—O1	11.2 (6)
C10—Si1—C1—C2	−141.2 (2)	C23A—O2—C22—C2	−168.3 (6)
C10—Si1—C1—C12	31.7 (2)		

### Ethyl 2,2-dimethyl-3-(naphthalen-1-yl)-1,2-dihydrobenzo[c]siline-4-carboxylate Hydrogen-bond geometry (Å, º)

**Table:**

*D*—H···*A*	*D*—H	H···*A*	*D*···*A*	*D*—H···*A*
C15—H15···O1^i^	0.93	2.49	3.359 (4)	156

Symmetry code: (i) −*x*+3/2, *y*+1/2, −*z*+1/2.
